# Machine-Learning-Assisted Quantitative Printability Assessment in Extrusion-Based Bioprinting—A Systems-Engineering Proof-of-Concept

**DOI:** 10.3390/bioengineering13091064

**Published:** 2026-09-13

**Authors:** Piotr Walecki, Anna Gula, Michał Rosowicz, Robert Żuk, Klaudia Proniewska-van Dam

**Affiliations:** 1 Department of Bioinformatics and Telemedicine, Medical College, Jagiellonian University, 30-688 Krakow, Poland; 2 Faculty of Management, AGH University of Krakow, 30-067 Krakow, Poland; gula@student.agh.edu.pl (A.G.); rosowicz@agh.edu.pl (M.R.); 3 Center for Digital Medicine and Robotics, Medical College, Jagiellonian University, 31-034 Krakow, Poland; robert.zuk@uj.edu.pl (R.Ż.); klaudia.proniewska@uj.edu.pl (K.P.-v.D.); 4 Functional and Virtual Medical 3D Imaging Laboratory, Department of Diagnostic Imaging, University Hospital in Krakow, 31-530 Krakow, Poland

**Keywords:** extrusion printing, extrusion bioprinting, printability, shape fidelity, image analysis, machine learning, process monitoring, systems engineering, open-architecture extrusion platform

## Abstract

Extrusion-based bioprinting is governed by coupled material, extrusion, and motion parameters, yet printability is often assessed using isolated rheological tests or qualitative geometric inspection. This study developed a systems-engineering framework for the quantitative assessment of non-cellular syringe-extrusion printing, with relevance to future bioprinting applications. Thirty-six constructs covered a complete 3 × 3 × 4 factorial design comprising three nozzle diameters, three printhead velocities, and four reference trajectories, with one independently printed construct per unique condition. Fiji/ImageJ analysis quantified filament width, edge roughness, curvature, and trajectory fidelity, and a study-relative Printability Score (PS) integrated four normalized geometric error domains. PS rankings were robust to moderate changes in component weighting (Spearman ρ = 0.955–0.999). PCA identified distinct deposition- and geometry-related modes; PC1, PC2, and PC3 explained 51.18%, 23.06%, and 11.69% of the variance, respectively (85.92% cumulative). Under LOOCV, raw-input Ridge Regression achieved $R^{2}$ = 0.713, MAE = 0.195, and RMSE = 0.285, whereas Gradient Boosting achieved $R^{2}$ = 0.709, MAE = 0.216, and RMSE = 0.288. Cross-validated permutation analyses identified printhead speed and trajectory geometry as the most informative raw predictors. These findings establish an offline engineering proof of concept rather than replicated confirmatory validation, biological validation, or closed-loop control.

## 1. Introduction

Extrusion-based three-dimensional (3D) bioprinting has become one of the most widely used biofabrication modalities because it can process a broad range of hydrogel-like materials, permits direct control of deposition trajectories, and is compatible with mechanically or pneumatically driven syringe systems [1,2,3]. In extrusion bioprinting, material is forced through a nozzle and deposited as a continuous filament for which its geometry is determined not by any single input variable but by the coupled behavior of the ink, dispensing system, motion platform, substrate, and stabilization strategy. This coupling is particularly important when the printed construct is expected to reproduce a computer-aided design with high dimensional fidelity.

Printability is therefore a central but variably defined concept in extrusion bioprinting. Depending on the study, the term has been used to describe extrudability, filament formation, shape fidelity, structural integrity, or combinations of these outcomes [4,5,6,7]. Recent work has argued for broader and more explicit definitions that link the ability to extrude a material with its ability to reproduce and retain the intended geometry [5,6,8]. For cell-laden bioinks, biological performance and cell viability add another dimension to the problem [8,9]. The present work deliberately focuses on the engineering component of printability—geometric fidelity and deposition stability under controlled extrusion conditions—before biological variability is introduced.

Rheological behavior is central to extrusion bioprinting. Viscosity, yield stress, shear-thinning behavior, viscoelastic recovery, and thixotropy affect nozzle flow, post-extrusion spreading, and the ability of a filament to retain its shape [6,10,11,12,13]. However, a material-centered definition is incomplete because the same ink can produce markedly different outcomes when nozzle geometry, pressure or piston motion, printhead velocity, acceleration, layer height, or path curvature changes [2,3,4,5,6]. Nozzle diameter and internal geometry alter flow resistance and shear conditions, whereas the relationship between material delivery and translational motion determines the amount of material deposited per unit path length [14,15]. Consequently, printability is better understood as an emergent response of the complete material–process–geometry system.

A second challenge is measurement. Much of the bioprinting literature relies on line tests, grid or lattice tests, pore-shape factors, strand-width measurements, or qualitative visual grading. These approaches are useful but are not always directly comparable across laboratories, materials, printers, and image-analysis procedures [7,10,16,17]. Standardization studies have shown that differences in equipment, calibration, preparation, and measurement protocols can produce substantial inter-laboratory variability even when nominally similar workflows are followed [16,17,18]. The need for standardized reference artifacts and transparent quantitative analysis has therefore become increasingly prominent [15,16,17].

Geometry itself can be used as an experimental variable rather than treated solely as the final object to be printed. Straight trajectories provide a low-dynamic-load baseline for extrusion uniformity; smooth curves introduce continuous changes in curvature; and sharp turns impose rapid changes in printhead velocity and acceleration, thereby revealing transient over-deposition, pressure lag, corner rounding, and delayed structural recovery. Printability artifacts and in situ printability maps have recently been proposed to reveal such relationships more systematically [15,19]. A structured family of trajectories can therefore function as an engineering stress test for the printing system.

At the same time, image analysis and machine learning (ML) provide a route from visual inspection to quantitative and reproducible process characterization. ML has already been used to accelerate parameter optimization in extrusion printing and to relate material and process variables to printing outcomes [9,20,21]. Deep-learning approaches have further demonstrated the feasibility of detecting extrusion defects and developing image-based quality-control loops [22]. Work on real-time flow sensing and dynamic pressure adjustment demonstrates the complementary potential of sensor-based control [18,23]. Nevertheless, many published workflows focus on material optimization, online defect classification, or individual process variables. There remains a practical need for well-structured datasets that integrate controlled process inputs, standardized geometry, and quantitative image-derived outputs within a single interpretable framework.

Previous quantitative approaches address complementary aspects of this problem. Paxton et al. linked rheological properties and lattice geometry to bioink printability [10], while Fu et al. reviewed quantitative concepts of extrudability, filament formation, and shape fidelity in extrusion bioprinting [5]. Gillispie et al. developed an artifact-based quantitative framework linking rheological properties with extrusion-based printability [15]. Strauß et al. and Grijalva Garces et al. emphasized standardized analytics, comparability, and cross-laboratory reproducibility [16,17]. Zanderigo et al. introduced in situ printability maps that relate operating settings to local deposition behavior [19], whereas Ruberu et al. demonstrated ML-assisted optimization of extrusion-printing conditions [20]. The contribution of the present study does not lie in proposing another isolated printability metric. Rather, it lies in integrating predefined CAD trajectories as controlled geometric stress tests, multi-domain image-derived error descriptors, a transparent study-relative composite score, and out-of-fold prediction from process-level variables within one compact factorial framework. This design is intended to complement rather than replace rheological, lattice-based, artifact-based, and in situ printability assessments.

For early-stage engineering calibration, a non-cellular model ink can be advantageous because it avoids confounding by cell concentration, viability, sterility constraints, biological degradation, and batch-to-batch variability. This does not make the model material biologically equivalent to a bioink, nor can conclusions about cell survival be inferred from it. Rather, it provides a controlled test medium for isolating mechanical and kinematic effects before the workflow is transferred to cell-laden systems. This distinction is particularly important in a study focused on platform characterization rather than biological validation.

Accordingly, this study aimed to develop and evaluate a system-level framework for engineering printability analysis that integrates (i) a custom open-architecture syringe-extrusion platform, (ii) a stable non-cellular model ink, (iii) a predefined reference design space spanning different geometric and kinematic demands, (iv) automated top-view image analysis, and (v) data-driven modeling of the relationship between process settings and geometric outcomes. We hypothesized that deposition scaling and trajectory geometry would explain printability more effectively than nozzle diameter alone and that a small set of interpretable geometric descriptors would capture distinct failure modes. The experiments characterize non-biological extrusion printing; references to extrusion-based bioprinting describe the broader application context rather than biological validation of the tested model-ink system. The demonstrated capability is offline quantitative characterization, whereas real-time multimodal sensing and closed-loop control are considered future extensions.

## 2. Materials and Methods

### 2.1. Custom Open-Architecture Syringe-Extrusion Platform

**Figure 1:**
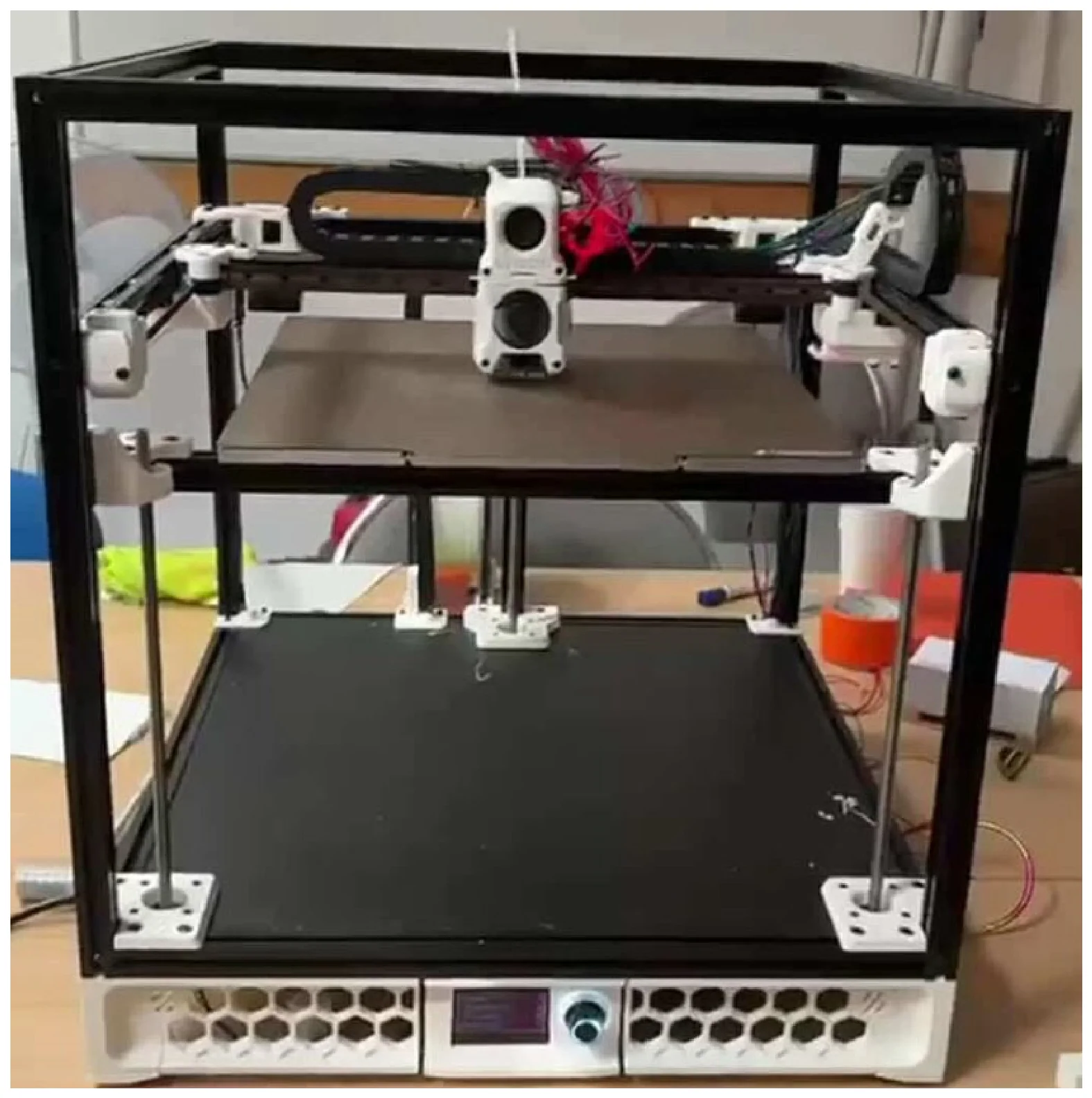
Custom open-architecture syringe-extrusion research platform based on a modified Voron Trident architecture (author-generated photograph).

A custom syringe-extrusion research platform was developed by adapting a Voron Trident CoreXY fused-deposition motion platform (Figure 1). The thermoplastic extrusion module was replaced with a mechanically driven syringe unit in which a stepper motor and screw-driven linear actuator advanced the syringe plunger. The mechanical drive was selected to provide consistent piston displacement and to avoid the compliance and pressure-storage effects typical of long pneumatic lines. The syringe assembly was supported by a rigid linear guide to minimize lateral plunger displacement and maintain axial alignment during extrusion. This open-architecture conversion strategy is consistent with previous work showing that desktop additive-manufacturing platforms can be adapted for low-cost syringe extrusion and biofabrication research [24,25,26].

The motion system retained the CoreXY kinematics and a vertically controlled build platform. Printer control was implemented using a Raspberry Pi 5, an Octopus control board, and Klipper firmware. A Sony IMX519 camera was integrated for process observation and recording. The syringe holder incorporated thermal regulation, and the material temperature during the reported experiments was maintained at 25–26 °C. The platform also included a 21-LED illumination module covering 365–405 nm (nominal total power 22 W, 24 V DC). In the present study, this illumination condition was held constant and was not analyzed as an independent experimental factor.

All test structures were deposited directly onto microscope glass slides. This minimized post-print handling before imaging and provided a planar, optically suitable substrate for subsequent top-view measurements. The platform was designed as an open, modular research system in which the extrusion unit, sensing, thermal control, illumination, and image-acquisition modules could be modified independently.

### 2.2. Non-Cellular Model Ink

A non-cellular physicochemical model ink was used during calibration and methodological development. The purpose of this model ink was not to reproduce the full biological behavior of a cell-laden hydrogel but to provide a stable test medium with consistent extrusion and post-deposition shape retention. The formulation was a multicomponent colloidal polymer system containing an aqueous/polar liquid phase with humectant components and a dispersed solid/polymeric phase containing mineral filler, pigments, film-forming polymers, rheology modifiers, plasticizing components, and surface-active additives.

The model ink was treated as an engineering test medium rather than as a rheologically validated bioink. No independent rheometer measurements were available for the present formulation; therefore, shear-rate-dependent viscosity, yield stress, storage and loss moduli, thixotropic recovery, and post-shear recovery cannot be reported retrospectively. No rheological value from another formulation was used as a surrogate measurement for the present model ink, and no literature-derived viscosity value was assigned to the material or used as an experimental variable. This distinction is important because hydrogel rheology can vary substantially with formulation and test conditions [27]; a value measured for another material cannot substitute for characterization of the present ink. Accordingly, interpretation is restricted to the observed geometric response of this specific non-cellular material under the tested conditions. Because the material contained no cells, no conclusions are drawn regarding cytocompatibility, cell viability, cell–material interactions, or biological functionality.

### 2.3. Reference Design Space and Geometric Stress Tests

Four predefined deposition trajectories were designed to impose distinct geometric and kinematic demands while sharing a nominal longitudinal extent of 50.00 mm and a nominal path width of 0.60 mm. Pattern A (stepped orthogonal zig-zag) comprised seven linear segments connected by six abrupt 90° directional changes, including three 12.50 mm vertical intervals and 20.00 mm horizontal excursions; it was intended to emphasize acceleration and deceleration, corner rounding, and local over-deposition. Pattern B (angular–curvilinear hybrid) combined linear and curved segments with nominal R = 8.00 mm arcs and an approximately 88.17° angular transition. Pattern C was a continuous-curvature/sinusoidal trajectory composed of successive nominal R = 8.33 mm arcs without discrete corners. Pattern D was a straight 50.00 mm reference line with no change in direction and served as the lowest-demand baseline.

The design rationale was to treat trajectory geometry as a controlled experimental input rather than to derive a universal scalar complexity index. The straight line minimizes curvature-dependent motion effects; the continuous-curvature pattern probes sustained path tracking; the hybrid path combines smooth curvature with a discrete transition; the stepped orthogonal path imposes repeated directional discontinuities. Geometry was therefore represented categorically in the primary models. Total path length was not used as an independent predictor, and the term “geometric demand” refers operationally to documented differences in curvature and directional discontinuity rather than to a validated universal complexity scale.

### 2.4. Experimental Design and Printing Conditions

**Figure 2:**
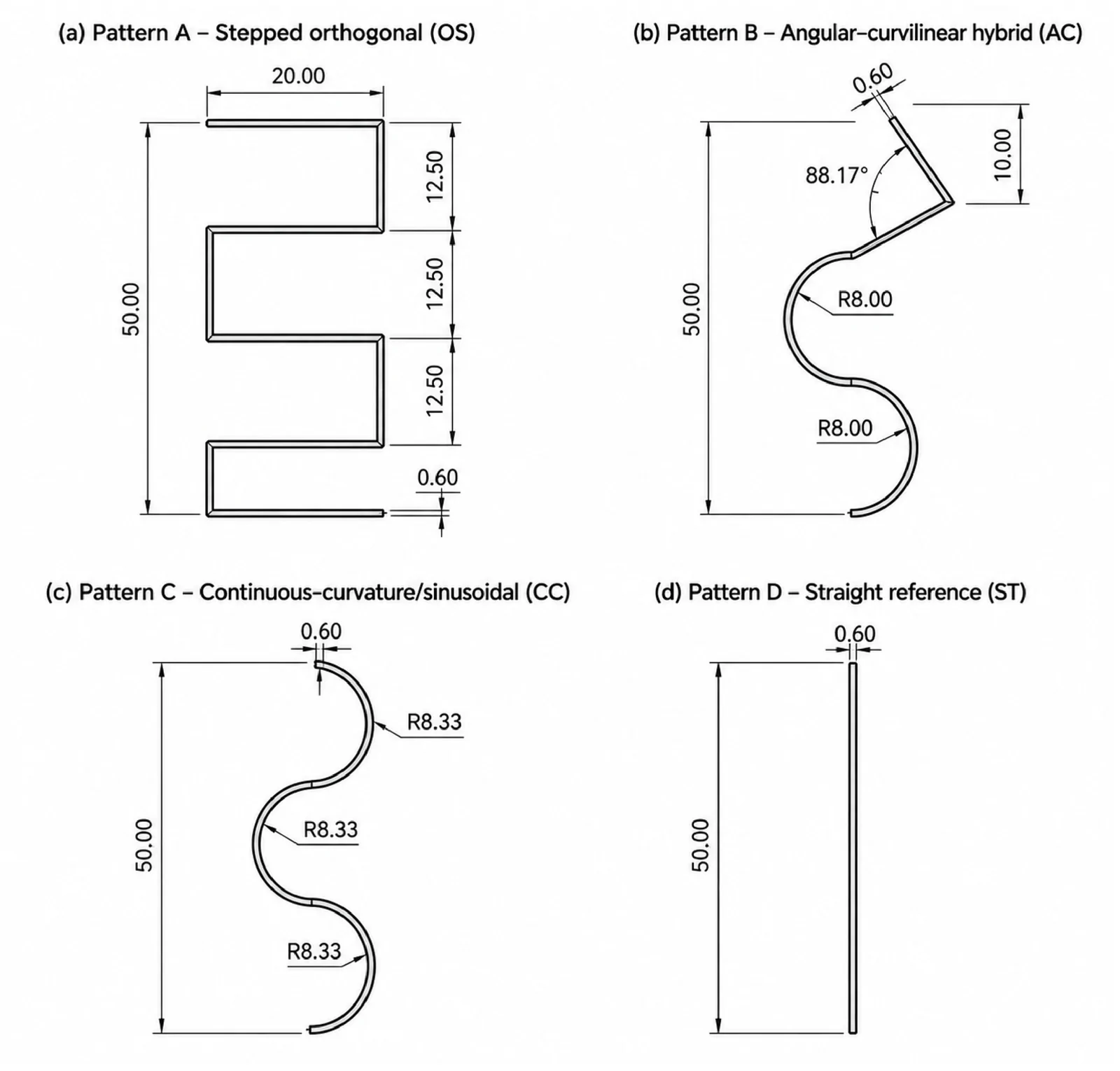
CAD-defined reference trajectories used as geometric stress tests in the extrusion-printing experiments. (**a**) Pattern A, stepped orthogonal trajectory (OS), incorporating successive 90° directional changes; (**b**) Pattern B, angular–curvilinear hybrid trajectory (AC), combining curved segments (R = 8.00 mm) with an approximately 88.17° angular transition; (**c**) Pattern C, continuous-curvature trajectory (CC), composed of successive curved segments with a nominal radius of R = 8.33 mm; and (**d**) Pattern D, straight reference trajectory (ST), representing the lowest-demand baseline. All trajectories had an overall longitudinal extent of 50.00 mm and a nominal path width of 0.60 mm.

The independent experimental unit was the printed construct. The design comprised 36 unique process–geometry combinations formed by three nozzle internal diameters, three printhead velocities, and four reference trajectories (Figure 2). One construct was printed for each unique combination. Multiple microscopic regions of interest (ROIs) sampled from the same construct were treated as repeated technical measurements and aggregated to construct-level descriptors before multivariate and machine-learning analyses; they were not treated as independent experimental replicates. Accordingly, the dataset provides complete factorial coverage for proof-of-concept modeling but no within-cell replication for confirmatory factorial inference.

The syringe plunger diameter was 12.6 mm, and the internal barrel diameter was 12.7 mm. Based on the barrel’s internal diameter, the nominal displaced-fluid cross-sectional area was 126.613 mm^2^. At a plunger advancement rate of 0.143 mm/s, the geometrically estimated nominal volumetric delivery rate was Q = 18.11 mm^3^/s. No synchronized inline flow sensor or independent gravimetric outlet-flow measurement was available during these experiments; Q therefore denotes the nominal kinematic delivery rate implied by piston displacement rather than a directly measured nozzle-exit flow rate. This distinction is retained throughout the analysis.

**Figure 3:**
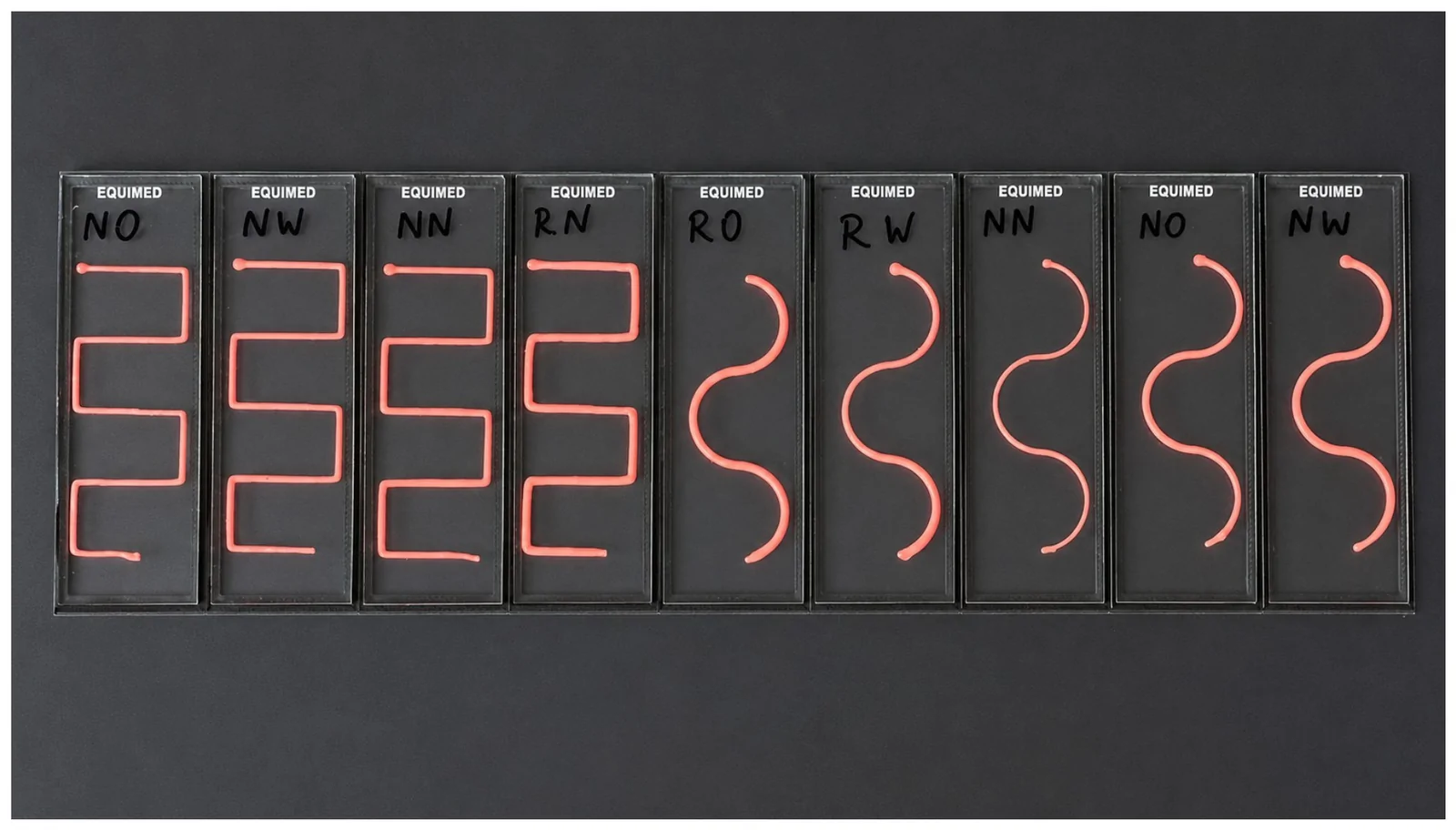
Representative model-ink constructs deposited on microscope glass slides during the extrusion-printing experiments. The photograph shows experimentally produced orthogonal and continuous-curvature trajectories before microscopic image acquisition and quantitative analysis. Multiple constructs are shown to demonstrate the physical realization of the programmed deposition patterns across selected experimental conditions.

Three blunt Luer-Lock dispensing nozzles were investigated: 0.4 mm internal diameter and 32 mm length, 0.6 mm internal diameter and 31 mm length, and 1.2 mm internal diameter and 32 mm length. The diameters were selected to provide a deliberately broad threefold range in outlet size while remaining within the mechanically stable operating range of the platform and model ink. Translational velocities of 14, 20, and 26 mm/s represented low, nominal, and high motion conditions (70%, 100%, and 130% of the nominal 20 mm/s setting) that maintained continuous deposition during platform commissioning. These levels were selected to create clearly separated engineering conditions rather than to define universal optimal bioprinting ranges. Nominal plunger advance was held constant while translational velocity varied, thereby changing material delivery per unit path length. The material’s temperature at the printhead was maintained at 25–26 °C for all runs (Figure 3).

Two derived quantities were used to describe process scaling. First, the nominal linear deposition load was defined as

$$A_{dep}=\frac{Q}{v}$$

where Q is the nominal volumetric delivery rate, and v is the printhead velocity. $A_{dep}$ has units of mm^2^ and represents the nominal deposited cross-sectional area implied by volume conservation. For Q = 18.11 mm^3^/s, the corresponding values were 1.294, 0.906, and 0.697 mm^2^ at 14, 20, and 26 mm/s, respectively. Second, the kinematic speed–nozzle ratio was defined as

$$R_{vD}=\frac{v}{D_{n}}$$

where $D_{n}$ is the nozzle internal diameter. $R_{vD}$ has units of s^−1^ and is therefore a scaling ratio rather than a dimensionless quantity. Because $A_{dep}$ is a deterministic transformation of speed when Q is held constant and $R_{vD}$ is derived from both speed and nozzle diameter, these engineered variables were treated as physically meaningful reparameterizations of the controlled design rather than as statistically independent causal predictors.

### 2.5. Image Acquisition and Standardized Segmentation

Because the model ink was optically opaque, printed structures were analyzed by standardized top-view reflected-light microscopy using a calibrated Nikon Eclipse E600 optical microscope (Nikon Corporation, Tokyo, Japan) equipped with a nominal 20-megapixel DLT-Cam PRO digital camera (Delta Optical, Warsaw, Poland). A fixed optical and illumination configuration was maintained throughout the experimental series. The ROI strategy was predefined according to trajectory geometry: stepped paths were sampled at internal and external corners, straight segments, and terminal regions; hybrid paths at the angular transition, line-to-curve interfaces, curved segments, and terminals; continuous-curvature paths at high-curvature, low-curvature, inflection, and terminal regions; and straight lines at the beginning, middle, and end. Approximately four to six images were collected per construct, and the construct—not the individual image—was the process-level analytical unit. The retained experimental records document the microscope model and nominal camera resolution but do not contain, for every ROI, the exact objective designation or magnification, exported pixel dimensions, exposure setting, numerical illumination setting, pixel-size calibration factor, or deposition-to-imaging interval. These unavailable metadata were not reconstructed retrospectively.

**Figure 4:**
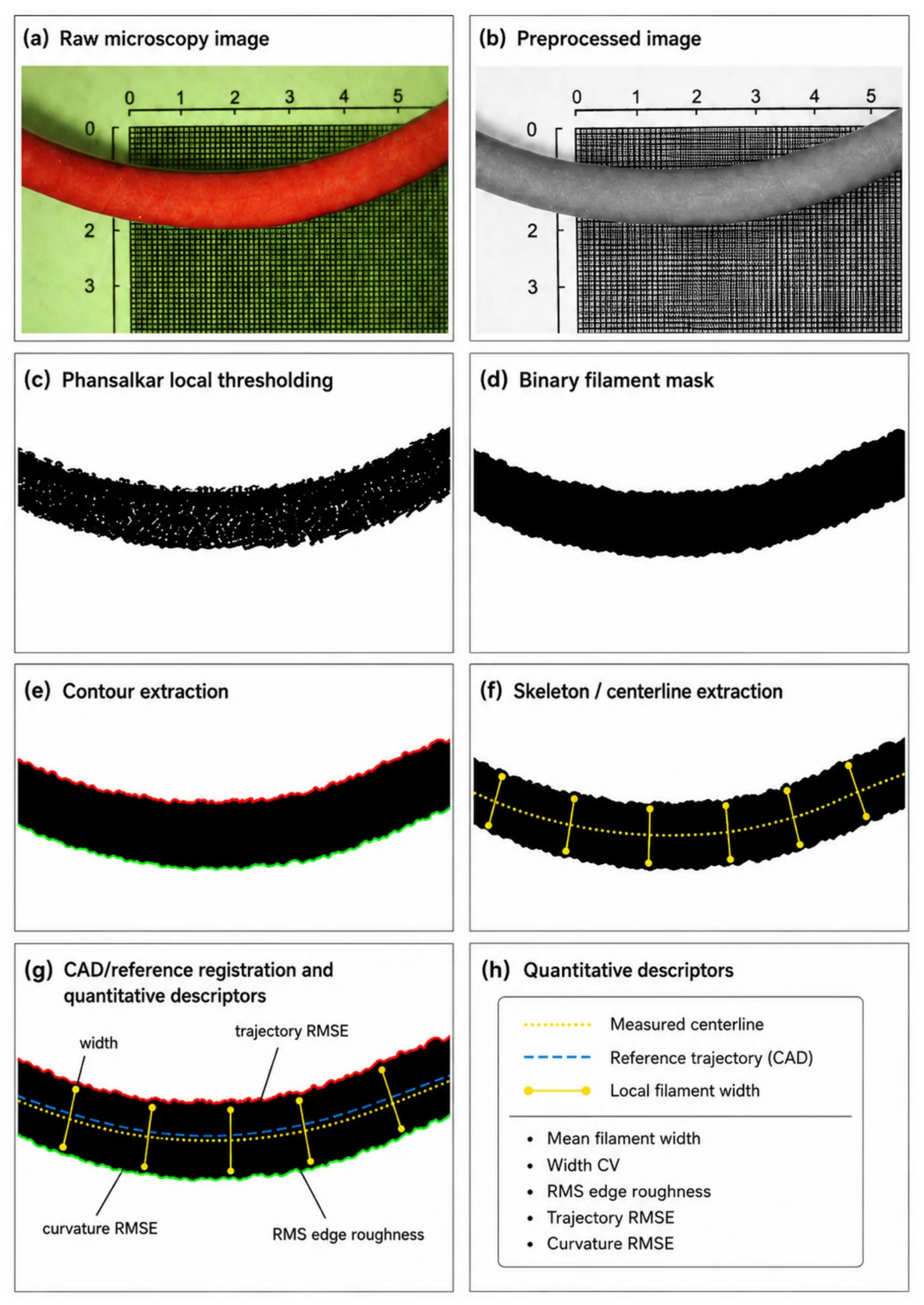
Schematic representation of the quantitative image-analysis workflow used for printability assessment. The diagram illustrates the principal computational stages applied to top-view microscopy images: (**a**) representative input image; (**b**) preprocessing, including background normalization and local contrast correction; (**c**) Phansalkar local thresholding; (**d**) morphological cleaning of the binary filament mask; (**e**) contour extraction for boundary analysis, with the upper and lower filament boundaries shown in red and green, respectively; (**f**) skeletonization and local filament-width determination; and (**g**) registration of the measured centerline to the corresponding CAD reference trajectory for geometric analysis. (**h**) The resulting descriptors included mean filament width, width CV, RMS edge roughness, trajectory RMSE, and curvature RMSE.

Images were processed in Fiji/ImageJ 1.54p 1.54p (National Institutes of Health, Bethesda, MD, USA) [28,29] using a standardized batch workflow comprising grayscale conversion, background normalization, local contrast correction, and noise reduction. Binary filament masks were generated using Phansalkar local thresholding and then subjected to morphological closing and removal of small isolated objects. The cleaned binary mask defined the filament boundary for contour-based analysis, while skeletonization generated the centerline used for width, trajectory, and curvature calculations. Spatial calibration applied during analysis converted length-based outputs to physical units. Binary masks, contours, skeletons, and registration overlays were generated for quality control, although the complete archive of intermediate images was not retained for every ROI. The current archive does not preserve the numerical Phansalkar parameters, morphological-kernel settings, or small-particle threshold used in the original batch workflow (Figure 4). These items are reported as unavailable rather than retrospectively inferred, and complete versioned macro-metadata are identified as a requirement for future replication studies. ROI-level measurements were aggregated to the construct level before correlation, PCA, and machine-learning analyses, thereby avoiding ROI-level pseudo-replication.

### 2.6. Quantitative Image-Derived Descriptors

All quantitative descriptors were derived from common intermediate representations of the segmented filament. A Euclidean distance transform was applied to the cleaned binary mask, and local filament widths were defined as twice the distance from the skeleton to the nearest mask boundary. Contours extracted from the cleaned binary filament mask were used for boundary analysis, while the skeletonized centerline was spatially registered to the corresponding CAD reference trajectory for positional and curvature comparisons. All length-based outputs were converted to physical units using the image-calibration factor applied during acquisition and analysis. The retained export contains the resulting registered descriptors but not the complete transformation and optimization settings used for CAD alignment; those settings cannot therefore be reconstructed reliably and are identified as metadata required for future replication.

Width-related descriptors comprised mean local width ($w_{mean}$), width standard deviation ($w_{SD}$), coefficient of variation (${CV}_{w}$), 95th-percentile width ($w_{95}$), and longitudinal width gradient ($\frac{dw}{ds}$). Boundary quality was summarized by RMS edge roughness (${RMS}_{edge}$), defined as the RMS deviation of the extracted contour from its smoothed representation. Geometric fidelity was characterized by mean centerline curvature ($\kappa_{mean}$), curvature RMSE relative to the registered reference path (${RMSE}_{curv}$), and centerline trajectory RMSE (${RMSE}_{traj}$). The end-bead ratio (EBR) and end-asymmetry index (EAI) were defined during workflow development as candidate endpoint descriptors; however, their construct-level values were not available in the locked 36-row analysis dataset. Accordingly, EBR and EAI were not included in the reported correlations, PCA, PS, or predictive models. The analyzed variables were retained because they represent complementary, physically interpretable failure modes rather than a single visual quality grade.

A composite Printability Score (PS) was used as a secondary continuous summary target. Four complementary instability metrics—width CV, trajectory RMSE, RMS edge roughness, and curvature RMSE—were min–max-normalized. For each component, normalization was defined as

$$x_{i}^{*}=\frac{x_{i}-x_{min}}{x_{max}-x_{min}}$$

The composite score was then calculated as

$$PS={CV}_{w}^{*}+{RMS}_{edge}^{*}+{RMSE}_{traj}^{*}+{RMSE}_{curv}^{*}$$

Higher PS values indicate poorer geometric printability. The score is explicitly study-relative: It is not a universal printability scale, and its numerical value depends on the normalization reference. Equal weighting was selected as the most transparent default in the absence of an externally validated basis for differential weighting. Robustness was therefore examined using leave-one-component-out scoring, halving and doubling individual component weights, rank-based normalization, robust 5th–95th percentile normalization, and random-weight sensitivity analysis. Primary interpretation remained anchored to the individual physical endpoints and the composite score. Detailed mathematical definitions of the process-level variables, image-derived descriptors, and the Printability Score are provided in Appendix A.

### 2.7. Statistical and Machine-Learning Analysis

All analyses were performed at the construct level (*n* = 36). Pairwise Pearson correlations among image-derived descriptors were used to characterize redundancy and coupled failure modes. Principal component analysis (PCA) was performed on nine standardized construct-level geometric descriptors: mean width, width SD, width CV, 95th-percentile width, longitudinal width gradient, RMS edge roughness, mean curvature, curvature RMSE, and trajectory RMSE. Eigenvalues, explained-variance ratios, correlation loadings, and variable contributions were reported for the leading components, and leave-one-construct-out jackknife analyses were used to assess component stability. Because there was one independent construct per unique factorial cell, ROI-level observations were not used to generate confirmatory *p*-values. Uncertainty was instead summarized descriptively using construct-level means and SDs, bootstrap intervals across balanced factor levels, and matched design-space contrasts that compared factor levels while holding the remaining design variables fixed. These intervals characterize robustness within the observed design space and are not substitutes for confidence intervals based on replicated factorial treatment effects.

Supervised regression was used to test whether PS could be predicted from process-level information without directly including the image-derived variables from which the score was constructed. Analyses used the 36-row construct-level dataset and were performed in Python 3.13.5 with scikit-learn 1.8.0. The primary predictor specification contained only the raw controlled inputs: nozzle diameter, printhead velocity, and categorical trajectory geometry. A secondary engineering specification additionally included $A_{dep}$ = $\frac{Q}{v}$ and $R_{vD}$ = $\frac{v}{D_{n}}$ to assess whether these deterministic reparameterizations improved prediction. The LOOCV training-set mean served as the naïve baseline. Ordinary Linear Regression, Ridge Regression (α = 1.0), a shallow Decision Tree Regressor, Random Forest Regression (RF), and Gradient Boosting Regression (GBR) were compared under leave-one-out cross-validation (LOOCV). Trajectory geometry was one-hot encoded without imposing ordinal spacing. For the linear and ridge models, numerical predictors were standardized within each training fold; tree-based models used unscaled numerical inputs. The decision-tree comparator used a maximum depth of 3 and a minimum leaf size of 2. RF used 500 trees, a maximum depth of 3, a minimum leaf size of 2, and sqrt feature sampling; GBR used 100 estimators, a learning rate of 0.05, a maximum depth of 2, and a minimum leaf size of 2. A random seed of 42 was used, and no hyperparameter search was performed.

**Figure 5:**
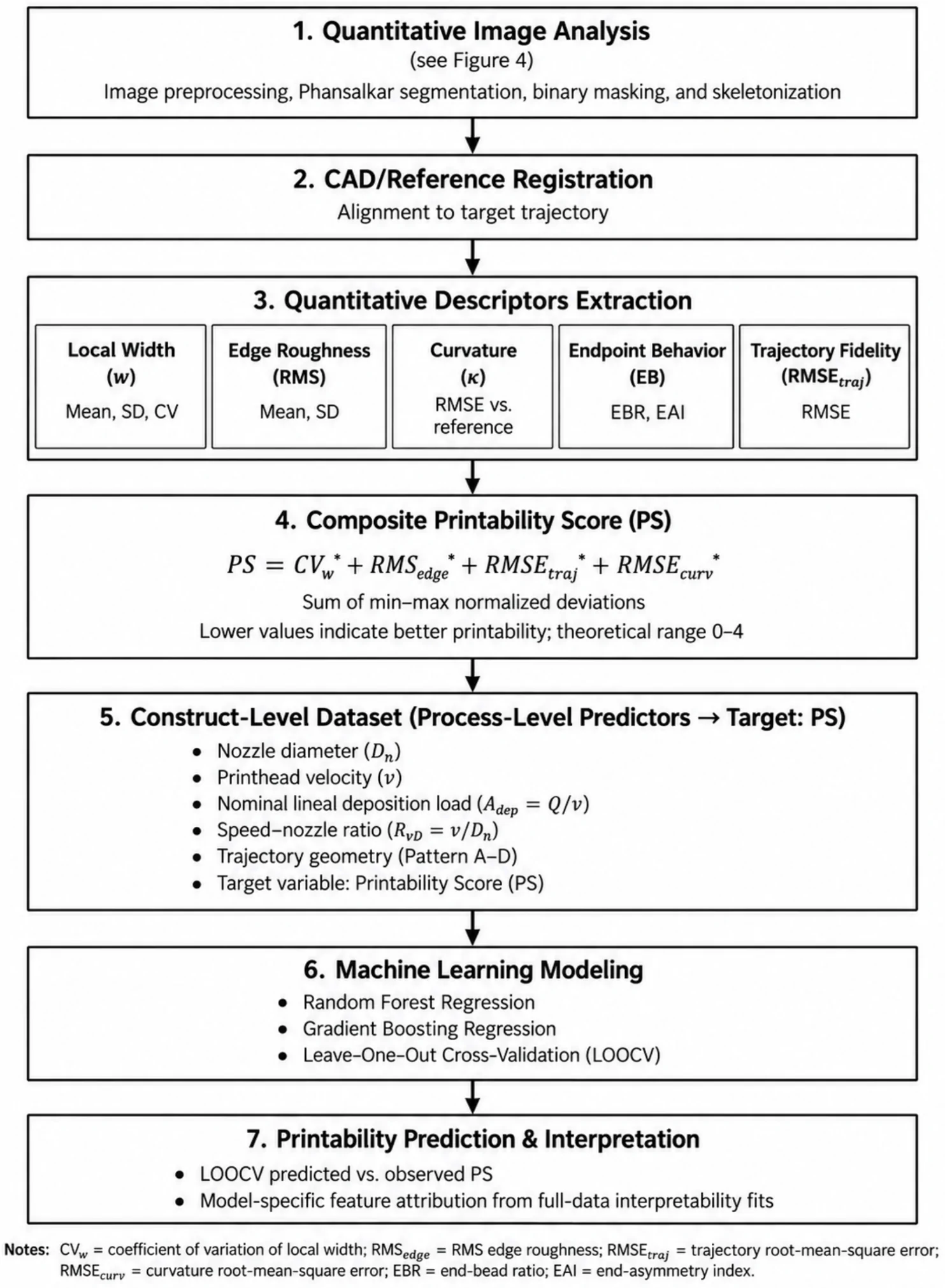
Integrated quantitative and machine-learning workflow for printability assessment. Image-derived geometric descriptors were registered to CAD reference trajectories and combined into a composite Printability Score (PS), with lower values indicating better geometric fidelity. The schematic shows both the raw controlled settings and the engineered reparameterizations $A_{dep}$ and $R_{vD}$. In the revised analysis, nozzle diameter, printhead velocity, and categorical trajectory geometry formed the primary predictor set, whereas $A_{dep}$ and $R_{vD}$ were evaluated secondarily. Linear Regression, Ridge Regression, and a shallow Decision Tree served as comparative baselines, with RF and GBR used as nonlinear ensemble comparators. Primary predictor relevance was assessed using cross-validated permutation importance and drop-one ablation. Endpoint behavior (EBR/EAI) is shown in the schematic as a candidate descriptor family, but endpoint values were not present in the final construct-level analytical table and were not used in PS, PCA, or ML. All predictive performance was evaluated using LOOCV.

All predictor-preprocessing steps were fitted within each LOOCV training fold and applied only to the corresponding held-out construct. Predictive performance was summarized from out-of-fold predictions using the coefficient of determination ($R^{2}$), mean absolute error (MAE), and root mean square error (RMSE); descriptive bootstrap intervals were calculated from the out-of-fold prediction pairs to characterize uncertainty in model error. Because no hyperparameter search was performed, the prespecified configurations were used identically in every fold. Predictor relevance was examined primarily through cross-validated permutation analysis of the raw controlled inputs and drop-one ablation, thereby avoiding misleading attributions among the algebraically related $A_{\mathrm{dep}}$, $R_{vD}$, speed, and nozzle terms. Full-data tree-native importances from the engineered specification were retained only as secondary descriptive checks and were not interpreted as causal effects. The min–max transformations defining PS were treated as part of the fixed study-level outcome definition rather than as predictor preprocessing; consequently, the reported LOOCV estimates concern the prediction of this study-relative target, and external deployment would require independently prespecified normalization bounds. The integrated quantitative and predictive workflow is summarized in Figure 5.

### 2.8. Use of Generative AI Tools

Generative AI tools were used for writing and computational support. ChatGPT (OpenAI; GPT-5.6 Sol) assisted with refinement of the ImageJ 1.54p and Python scripts. All code was executed on the authors’ data, and all outputs were reviewed and verified by the authors. Generative AI was not used to generate experimental measurements, microscopy-derived data, or the underlying experimental observations.

## 3. Results

### 3.1. Process-Dependent Filament Morphology and Geometric Fidelity

Across the 36 construct-level observations, filament widths varied systematically with nozzle diameter and translational speed. The mean filament width ranged from approximately 720 to 1260 µm for the 0.4 mm nozzle, 760 to 1550 µm for the 0.6 mm nozzle, and 1250 to 2400 µm for the 1.2 mm nozzle. At a fixed nominal plunger advance, higher printhead velocity was associated with narrower filaments across nozzle sizes and trajectory types, consistent with the lower nominal lineal deposition load, $\frac{Q}{v}$.

Width variability increased most strongly for the 1.2 mm nozzle under low-speed conditions, where the coefficient of variation reached approximately 0.32–0.38 depending on the trajectory. Pattern A showed the greatest local variability near abrupt directional transitions, with frequent corner thickening. Pattern B showed intermediate behavior, whereas Pattern C was generally more uniform than the discontinuous geometries. Pattern D, the straight reference trajectory, consistently showed the lowest width variance and therefore provided the most stable baseline for intrinsic spreading and extrusion variation.

RMS edge roughness showed a similar descriptive pattern. Values for the 0.4 mm nozzle were approximately 68–260 µm depending on speed and trajectory, whereas values above 300 µm were observed for the 1.2 mm nozzle in more demanding trajectories. Roughness generally decreased as printhead velocity increased within the tested range. Pattern A showed the highest roughness overall, whereas Pattern D showed the lowest roughness. These comparisons describe construct-level patterns and are not presented as replicated factorial significance tests.

Curvature and trajectory errors were also geometry-dependent. Curvature RMSE generally declined with increasing printhead speed, but one severe curvature-specific failure occurred for A_N0.4_V14, for which the recorded curvature RMSE was 96.000 mm^−1^; all other construct-level curvature RMSE values were ≤0.478 mm^−1^. This verified value was retained in all primary analyses, and its influence was examined explicitly using PCA jackknife analysis and PS sensitivity analysis. Trajectory RMSE was highest under low-speed conditions for the more demanding patterns and exceeded 300 µm in selected Pattern A and Pattern C conditions. The straight reference line showed the smallest trajectory deviations, with minimum values below 60 µm under high-speed conditions. Taken together, the descriptive results indicate a consistent operating tendency: greater nominal material delivery per unit path length was associated with wider filaments, rougher boundaries, and poorer trajectory fidelity, while trajectory discontinuities amplified localized errors.

Matched design-space contrasts quantified the consistency and magnitude of these tendencies without treating repeated ROIs as independent replicates. Across the 12 matched nozzle × geometry strata, PS was higher at 14 than at 26 mm/s in all 12 comparisons, with a mean difference of 0.745 (SD: 0.237; descriptive bootstrap 95% interval 0.636–0.887). The 1.2 mm nozzle produced a higher PS than the 0.4 mm nozzle in 11 of 12 matched speed × geometry strata (mean difference: 0.407; SD: 0.218; 95% interval: 0.282–0.512). Relative to straight Pattern D, Pattern A showed a higher PS in all nine matched nozzle × speed strata (mean difference: 0.821; SD: 0.292; 95% interval: 0.665–1.022), as did Pattern B (mean difference: 0.590; SD: 0.093; 95% interval: 0.535–0.649). At constant Q, slopes relating each outcome to $A_{dep}$ were positive in all 12 nozzle × geometry strata for mean width, RMS edge roughness, trajectory RMSE, and PS; the mean PS slope was 1.237 per mm^2^ of $A_{dep}$ (descriptive bootstrap 95% interval 1.053–1.470). These intervals describe robustness across the balanced design space and are not confirmatory factorial confidence intervals. Accordingly, the contrasts quantify consistency within the sampled design space and should not be interpreted as inferential evidence for population-level treatment effects.

### 3.2. Correlation Structure and Principal Components

The construct-level image descriptors showed a highly structured correlation pattern. Mean width, width standard deviation, 95th-percentile width, and width coefficient of variation were strongly correlated (r = 0.886–0.984), indicating that filament expansion and width instability formed a common morphological axis. Edge roughness was also strongly associated with the width variables (r ≈ 0.81). Trajectory RMSE and curvature RMSE showed a moderate overall correlation (r = 0.384), but this association was strongly influenced by the verified curvature-specific extreme at A_N0.4_V14; omitting that construct in a sensitivity analysis reduced the correlation to r = 0.039. Thus, the two descriptors captured largely distinct error modes across most of the investigated design space, while sharing information under the most severe curvature failure. Selected pairwise correlations are summarized in Table 1.

**Figure 6:**
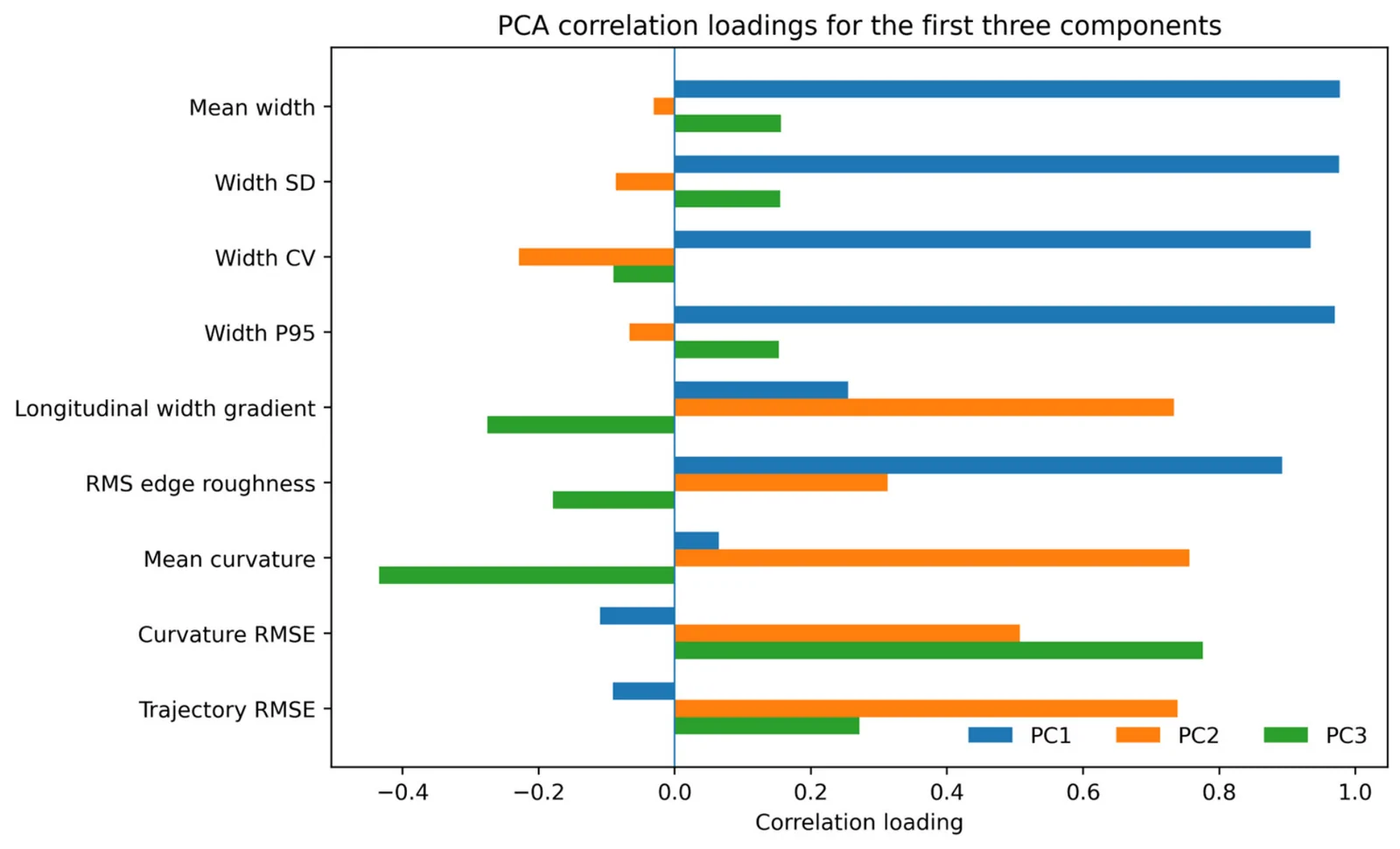
Correlation-loading plot for the first three principal components based on the 36-construct dataset. Positive and negative values indicate the direction and magnitude of the association between each standardized descriptor and the corresponding component. PC1 is dominated by width and edge-roughness variables, PC2 by trajectory and dynamic variables, and PC3 by curvature RMSE.

PCA supported this multidimensional interpretation. PC1 had an eigenvalue of 4.606 and explained 51.18% of the total variance; it was dominated by filament-width and edge-roughness descriptors, consistent with a deposition-driven instability mode. PC2 had an eigenvalue of 2.075 and explained 23.06%; its strongest correlation loadings involved mean curvature, trajectory RMSE, and longitudinal width gradient, reflecting geometry-dependent tracking and dynamic response. PC3 had an eigenvalue of 1.052, explained 11.69% of the variance, and was dominated by curvature RMSE. Together, the first three components explained 85.92% of the total variance. The leading-component loading structure is summarized in Table 2 and visualized in Figure 6. Leave-one-construct-out jackknife analysis showed comparatively stable PC1 and PC2 solutions, whereas PC3 was more sensitive to omission of individual constructs because the verified A_N0.4_V14 curvature failure contributed strongly to this curvature-specific mode. PCA is interpreted as descriptive dimensionality reduction rather than population-level latent-variable inference.

### 3.3. Prediction of Composite Printability from Process Parameters

Model comparison under identical LOOCV showed that predictive performance did not depend on a complex ensemble model. The training-mean baseline yielded $R^{2}$ = −0.058, MAE = 0.457, and RMSE = 0.548. Using only the raw controlled inputs, Linear Regression achieved $R^{2}$ = 0.712, MAE = 0.198, and RMSE = 0.286; Ridge Regression achieved $R^{2}$ = 0.713, MAE = 0.195, and RMSE = 0.285; a shallow Decision Tree achieved $R^{2}$ = 0.425, MAE = 0.301, and RMSE = 0.404; RF achieved $R^{2}$ = 0.541, MAE = 0.290, and RMSE = 0.361; and GBR achieved $R^{2}$ = 0.709, MAE = 0.216, and RMSE = 0.288. Descriptive bootstrap 95% RMSE intervals were 0.199–0.363 for Linear Regression, 0.197–0.363 for Ridge, 0.278–0.441 for RF, and 0.212–0.355 for GBR. The overlap among the strongest models argues against over-interpreting small numerical differences. A secondary specification that added $A_{dep}$ and $R_{vD}$ yielded $R^{2}$ = 0.730 for Ridge (MAE = 0.195; RMSE = 0.277), 0.511 for RF (MAE = 0.308; RMSE = 0.373), and 0.677 for GBR (MAE = 0.234; RMSE = 0.303). Thus, simple linear and regularized models matched or slightly outperformed the nonlinear ensembles, indicating that much of the process–printability relationship in this design was structured and comparatively low-dimensional. Because $A_{dep}$ and $R_{vD}$ are deterministic transformations of the controlled settings, the parsimonious raw-input specification was used for primary interpretation. The corresponding LOOCV model-performance comparison is summarized in Table 3.

### 3.4. Model Interpretation

Cross-validated interpretability analyses were performed using the raw controlled inputs to avoid repeatedly partitioning the same information among speed, $A_{dep}$, $R_{vD}$, and nozzle diameter. In the raw-input GBR model, permuting printhead speed increased out-of-fold RMSE by 0.204 on average (descriptive bootstrap 95% interval 0.128–0.273), permuting trajectory geometry increased RMSE by 0.203 (0.120–0.278), and permuting nozzle diameter increased RMSE by 0.141 (0.078–0.200). Ridge Regression showed the same ordering, with corresponding mean RMSE increases of 0.221, 0.197, and 0.118. Thus, printhead speed and trajectory geometry provided the greatest predictive information in the parsimonious models, while nozzle diameter contributed additional independent information. Cross-validated permutation importance for the raw controlled predictors is summarized in Table 4.

Drop-one ablation provided convergent evidence. For the raw-input GBR model, removing printhead speed reduced $R^{2}$ from 0.709 to 0.221, removing trajectory geometry reduced $R^{2}$ to 0.260, and removing nozzle diameter reduced $R^{2}$ to 0.363. The corresponding ridge values were 0.300, 0.382, and 0.521, respectively, compared with $R^{2}$ = 0.713 for the full raw-input model. These cross-validated results support substantial roles for both motion and trajectory geometry. The separate $A_{dep}$ analysis was directionally consistent: greater nominal material delivery per unit path length was associated with higher PS across all 12 matched nozzle × geometry strata.

### 3.5. Robustness of the Composite Score and Component-Specific Prediction

The equal-weight PS was robust to moderate changes in weighting and normalization. Halving or doubling any single component weight strongly preserved the construct ranking (Spearman ρ = 0.955–0.999). Leave-one-component-out rankings remained highly concordant (ρ = 0.865–0.965), with the largest change observed when trajectory RMSE was omitted; removing the curvature-RMSE component still yielded ρ = 0.965. Rank/percentile normalization and robust 5th–95th percentile normalization produced ρ = 0.950 and 0.952, respectively, relative to the primary score. Across 20,000 random weight vectors, the median rank correlation with the equal-weight PS was 0.920 (5th–95th percentile 0.714–0.989). These analyses indicate that the overall ordering does not depend on a narrowly tuned weighting choice, while confirming that PS remains a study-relative composite rather than a universal scale.

As an internal consistency check, PS also showed positive associations with image descriptors that were not components of the score, including mean width (Pearson r = 0.727), width SD (r = 0.708), 95th-percentile width (r = 0.687), longitudinal width gradient (Spearman ρ = 0.750), and mean curvature (r = 0.349). These relationships support convergent interpretation but do not constitute independent criterion validation because all descriptors originated from the same imaging workflow. Component-specific LOOCV models further showed that trajectory RMSE and edge roughness were strongly predictable from the raw process settings (GBR $R^{2}$ = 0.858 and 0.817, respectively), whereas the curvature-RMSE component was not (GBR $R^{2}$ = −0.784), reflecting the highly localized A_N0.4_V14 curvature failure. Importantly, the total PS ranking remained stable when the curvature component was removed (ρ = 0.965), demonstrating that the principal composite conclusions did not depend on that single mode. Independent validation against established printability artifacts and/or blinded expert assessment remains necessary.

## 4. Discussion

### 4.1. Integrating the Findings into a System-Level Framework of Printability

The central finding of this study is that geometric printability in the investigated extrusion system is better understood as a coupled material–process–geometry response than as a property of nozzle diameter or model-ink behavior alone. Four lines of evidence support this interpretation. First, image-derived morphology changed systematically with nominal material delivery per unit path length and trajectory geometry. Second, matched design-space contrasts showed highly consistent speed and geometry effects despite the absence of within-cell replication. Third, PCA separated a dominant width/edge-instability mode from tracking- and curvature-related modes, with the first three components accounting for 85.92% of the total variance. Fourth, multiple internally cross-validated regression models predicted PS substantially better than the mean baseline, with parsimonious raw-input ridge and GBR models each explaining approximately 71% of the variance. The PS sensitivity analysis further showed that these conclusions did not depend on a narrowly tuned weighting scheme.

This system-level perspective is consistent with the current printability literature, which increasingly distinguishes extrudability, filament fidelity, and structural accuracy while emphasizing interactions among ink properties, printer settings, and construct design [4,5,6,7,8]. Paxton et al. linked printability to rheology and lattice geometry [10]; Gillispie et al. developed an artifact-based quantitative assessment framework [15]; Strauß et al. and Grijalva Garces et al. emphasized standardized analytics and cross-laboratory reproducibility [16,17]; Zanderigo et al. introduced in situ printability maps [19]; Ruberu et al. demonstrated ML-assisted extrusion optimization [20]. The present contribution is complementary: It combines CAD-defined trajectory stress tests, multi-domain image-derived fidelity measures, an explicitly study-relative composite target, and process-only out-of-fold prediction within one compact factorial framework. Its novelty therefore lies in system-level integration and the separation of geometry-sensitive failure modes rather than in replacing established rheological, lattice, artifact-based, or in situ methods.

### 4.2. Deposition Scaling, Printhead Velocity, and Nozzle Effects

Within the tested range, increasing printhead velocity while maintaining nominal piston advance reduced filament width, edge roughness, trajectory error, and the composite PS. This behavior is physically plausible because the nominal lineal deposition load, defined here as $\frac{Q}{v}$, decreases as translational speed increases. The matched analysis strengthened this interpretation: PS was higher at 14 than at 26 mm/s in all 12 nozzle × geometry strata, and slopes relating mean width, RMS edge roughness, trajectory RMSE, and PS to $A_{dep}$ were positive in all 12 strata. Under over-deposition conditions, a larger volume of material must be accommodated per unit path length, promoting lateral spreading and increasing the geometric extent over which boundary irregularities can develop. Similar dependencies of strand width on flow rate, speed, nozzle geometry, and ink rheology have been reported in experimental and computational studies of extrusion bioprinting [2,3,4,5,6,9,14].

The cross-validated importance analysis provides a more interpretable separation of the controlled inputs than tree-native attribution based on algebraically related engineered variables. Permutation and drop-one analyses identified printhead speed and trajectory geometry as the strongest raw predictors, while nozzle diameter provided a smaller but still substantive independent contribution. This does not imply that nozzle physics is secondary in a mechanistic sense: nozzle diameter affects flow resistance, shear conditions, extrusion pressure, and achievable spatial resolution [9,14]. Rather, within the present three-level design, motion and geometric demand accounted for more of the predictable between-construct variation in PS. The engineered $A_{dep}$ and $R_{vD}$ terms remain useful for physical interpretation, but they did not improve the cross-validated performance of RF or GBR and were therefore treated as secondary reparameterizations rather than independent causal variables.

### 4.3. Reference Geometry as an Engineering Stress Test

The four trajectories were selected to impose different kinematic demands and to extend the analysis beyond isolated straight-line or lattice tests. The straight pattern provided the most stable baseline, whereas abrupt turns accentuated width variation and edge roughness. Smooth curvature introduced different errors, including path smoothing and curvature deviation. This separation is useful because similar low-fidelity prints can arise from different mechanisms, including over-deposition, transient pressure lag, motion-system tracking error, delayed material recovery, or endpoint dynamics.

The broader field has recognized the need for standardized artifacts and comparable quantitative protocols [7,15,16,17]. The round-robin study by Grijalva Garces et al. demonstrated that reproducibility across laboratories remains challenging even under harmonized procedures [17], while Gillispie et al. developed a dedicated artifact linking rheology with printability outcomes [15]. Zanderigo et al. introduced in situ printability maps as a framework for relating process settings to measurable printing behavior [19]. The present reference design space is complementary to these approaches: it is intended not to replace established lattice or rheological tests but to provide a compact trajectory set that distinguishes baseline deposition from curvature- and acceleration-sensitive error modes.

### 4.4. AI-Assisted Quantification and Relevance to Process Monitoring

The predictive component of this study should be interpreted as an exploratory engineering layer built on standardized quantitative outcomes, not as a substitute for physical understanding. With only 36 construct-level observations, model complexity must be constrained, and performance should be interpreted conservatively. The reviewer-requested baseline comparison was therefore informative: Linear and Ridge Regression using only nozzle diameter, printhead speed, and categorical geometry achieved LOOCV $R^{2}$ values of 0.712 and 0.713, respectively, while raw-input GBR achieved $R^{2}$ = 0.709. The similar performance of simple linear, regularized, and nonlinear models suggests that the dominant process–printability relationship in this design is structured and largely low-dimensional rather than dependent on a highly flexible learner. Thus, the observed predictive performance should not be interpreted as evidence of an intrinsic advantage of machine learning over simpler predictive models in this small dataset. A secondary engineered Ridge specification reached $R^{2}$ = 0.730, but this numerical improvement should not be over-interpreted because $A_{dep}$ and $R_{vD}$ are deterministic transformations of the controlled settings. These results represent internal cross-validation and do not establish transportability across materials, platforms, or laboratories. Recent work in Bioengineering has likewise demonstrated the value of coupling physical models with AI-driven image analysis to estimate state variables that are difficult to recover from deterministic modeling alone [30]. Although the present study addresses a different process, the underlying measurement logic is complementary: deposited geometry is treated as an observable state of the extrusion system rather than as a purely visual endpoint.

Previous studies have shown that ML can accelerate optimization of extrusion-printing conditions [20], while deep-learning systems can classify defects and support image-based quality-control loops [22]. Sensor-based studies have independently demonstrated real-time flow monitoring and dynamic pressure adjustment [18,23]. The current workflow occupies an intermediate position: It transforms post-print images into objective quantitative descriptors and links those descriptors to process settings. This positions the workflow along the path toward multimodal AI-assisted bioprinting, although the present implementation remains offline. A logical next step is to replace or supplement post-print microscopy with synchronized camera, force/pressure, and flow-rate signals so that the same geometric error modes can be detected during deposition and ultimately incorporated into closed-loop control.

The non-cellular model ink was used to isolate engineering variability during platform development. This strategy reduces costs and avoids biological confounders such as cell concentration, viability, sterility, culture time, and biologically induced changes in material behavior. For calibration, this is advantageous because observed failures can be attributed more directly to the coupled effects of motion, nominal delivery, trajectory geometry, and image analysis.

However, the model material does not provide biological validation of the system, and the absence of full rheological characterization materially limits generalizability. Shear-rate-dependent viscosity, yield stress, storage and loss moduli, thixotropic recovery, and post-shear structural recovery were not measured for the present material. These properties can influence nozzle flow, die swell, filament spreading, corner behavior, structural recovery, and the stresses experienced by suspended cells [6,10,11,12,13]. Cell-laden hydrogels additionally introduce concentration-dependent rheology, cell–matrix interactions, viability constraints, gelation and crosslinking kinetics, swelling, and sensitivity to temperature or irradiation [6,8,9,11,12,13]. Consequently, the process relationships reported here are conditional on this non-cellular model ink and cannot be assumed to transfer quantitatively to other bioinks or cell-laden formulations without new rheological and biological validation.

### 4.5. Limitations and Future Work

This study has several limitations. First, the 36 constructs represent one print for each unique combination of nozzle diameter, speed, and trajectory. The design therefore provides broad factorial coverage but no independent replication within individual factorial cells. This limits conventional variance estimation and precludes strong confirmatory significance claims. The matched contrasts and bootstrap intervals reported here characterize consistency and uncertainty within the observed design space; they do not replace independent experimental replication and should not be interpreted as population-level treatment-effect inference. Future experiments should include independently prepared and printed replicates, randomized run order, and, where appropriate, hierarchical models that distinguish construct-level variation from ROI-level measurement variation.

Second, only one non-cellular model ink, one mechanically driven extrusion mechanism, one substrate type, and one custom platform were evaluated. The quantitative relationships should not be assumed to generalize unchanged across hydrogel formulations, cell concentrations, nozzle materials and geometries, pneumatic or other extrusion mechanisms, temperatures, substrates, crosslinking conditions, or commercial printing platforms. External validity should therefore be tested across these dimensions using independently prepared constructs.

Third, comprehensive rheological characterization of the model ink was not available. Future transfer studies should include shear-rate-dependent viscosity, yield behavior where relevant, oscillatory storage and loss moduli, recovery or thixotropy, measurement temperature, measurement geometry, and independent material replicates. A literature value from another hydrogel cannot substitute for these measurements. Similarly, the present Q is a nominal kinematic delivery rate derived from piston displacement; future studies should include synchronized gravimetric or inline flow measurements to quantify actual outlet flow and transient delivery.

Fourth, the composite Printability Score is study-relative. It sums four min–max-normalized error metrics with equal weighting, which is transparent but not unique. Sensitivity analysis showed strong rank stability under moderate weight changes (ρ = 0.955–0.999) and under rank-based or robust percentile normalization (ρ ≈ 0.95), but this does not constitute external criterion validation. The verified A_N0.4_V14 curvature-RMSE value of 96.000 mm^−1^ demonstrates that a severe localized failure can strongly influence one normalized component and the curvature-specific PCA mode. Importantly, removing the curvature component preserved the total PS ranking closely (ρ = 0.965). Future work should validate PS against independent expert assessment or established printability artifacts and define fixed external calibration bounds before deployment.

Fifth, the engineered predictors are not statistically independent. Nominal lineal deposition load is a direct function of flow and speed, and the speed–nozzle ratio is a direct function of speed and nozzle diameter. The primary models therefore use raw controlled inputs, while $A_{dep}$ and $R_{vD}$ are treated as secondary engineering reparameterizations. Cross-validated permutation importance and drop-one ablation, rather than full-data tree-native importance, provide the main evidence for predictor relevance. Even with this conservative strategy, the dataset remains small, and LOOCV provides internal rather than external validation. Independent constructs produced in separate printing sessions and, ultimately, with different materials and platforms are required before the predictive models can be considered externally validated.

Sixth, although a fixed microscope and illumination configuration was used, complete retrospective acquisition and macro-metadata were not preserved at the level required for an exact reproduction of every image-processing step. The retained records document the microscope model, nominal camera resolution, workflow sequence, and use of Phansalkar thresholding but not the exact objective designation for every ROI, exported pixel dimensions, exposure settings, pixel-size calibration factor, numerical Phansalkar parameters, morphological-kernel settings, particle-removal threshold, or complete CAD-registration transformation and optimization settings. Future studies should archive these metadata, spatial calibration (µm/pixel), versioned Fiji/ImageJ macros, and ROI-level source measurements. This is particularly important for curvature and boundary metrics, which are sensitive to spatial resolution, segmentation, and skeletonization details.

Finally, image acquisition was performed after deposition. A key direction for translation is multimodal synchronization of top-view or coaxial imaging with piston force, pressure, flow rate, temperature, and machine kinematics. Such data would allow temporal alignment between a local geometric defect and the process event that preceded it and would provide a more direct basis for closed-loop control. More broadly, image-based 3D modeling and additive manufacturing have demonstrated the ability to reproduce complex anatomical structures with sufficient geometric fidelity for educational and clinical applications [31]. This direction is consistent with the broader convergence of 3D modeling, additive manufacturing, immersive visualization, and artificial intelligence that is increasingly shaping digitally assisted medical practice [32]. The present work, however, demonstrates an offline quantitative framework rather than a real-time feedback controller. The next validation stage should combine independently replicated printing runs, direct rheological characterization of the actual test material, independent measurement of extrusion flow, and an external test set generated in a separate printing session before extension to cell-laden bioinks or claims of generalizable predictive performance.

## 5. Conclusions

This study presents a non-cellular systems-engineering proof of concept for quantitative syringe-extrusion printability assessment relevant to extrusion-based bioprinting. By combining a custom open-architecture platform, a model ink, controlled trajectory geometries, standardized image-derived descriptors, and cross-validated predictive modeling, the approach captures printability as a coupled response to deposition scaling and geometric demand. Across 36 process–geometry conditions, increased nominal lineal deposition load and more demanding trajectories were associated with greater filament expansion, edge irregularity, trajectory error, and higher PS; the 14-versus-26 mm/s PS contrast was positive in all 12 matched nozzle × geometry strata. PCA identified separable deposition- and geometry-related failure modes, with PC1–PC3 explaining 85.92% of the total variance. Under LOOCV, parsimonious raw-input Ridge Regression achieved $R^{2}$ = 0.713 (MAE = 0.195; RMSE = 0.285), while Gradient Boosting achieved $R^{2}$ = 0.709 (MAE = 0.216; RMSE = 0.288), indicating substantial predictable structure without requiring a uniquely complex model. Cross-validated permutation and ablation analyses identified printhead speed and trajectory geometry as the strongest raw predictors. The composite score remained robust to moderate changes in weighting and normalization. The current evidence is intentionally limited to offline, non-biological, internally cross-validated engineering characterization based on one construct per factorial condition. Independent experimental replication, direct rheological characterization, independent measurement of actual extrusion flow, complete imaging and macro-metadata, an external test set from a separate printing session, and subsequent testing with cell-laden bioinks are required before generalizable, biologically predictive, or closed-loop bioprinting claims can be made.

## Figures and Tables

**Table 1:** Correlation structure of selected image-derived descriptors (construct-level data, *n* = 36).

Feature Pair	Pearson r	Interpretation
$w_{mean}$–$w_{95}$	0.984	Strong redundancy; both describe filament-width scale
$w_{mean}$–$w_{SD}$	0.982	Width dispersion is strongly coupled to mean width
$w_{SD}$–${CV}_{w}$	0.916	Relative variability is strongly associated with width dispersion
$w_{mean}$–${CV}_{w}$	0.886	Larger filaments show greater relative width variability
${RMS}_{edge}$–$w_{mean}$	0.818	Boundary roughness increases with filament expansion
${RMS}_{edge}$–$w_{SD}$	0.808	Roughness is coupled to the amplitude of width instability
${RMSE}_{traj}$–${RMSE}_{curv}$	0.384	Partial independence of trajectory tracking and curvature fidelity
${CV}_{w}$–$\frac{dw}{ds}$	0.079	Longitudinal width gradient captures a distinct variation mode

**Table 2:** Principal component analysis based on the verified 36-construct dataset. Loadings are correlations between standardized descriptors and component scores.

Principal Component	Eigenvalue/ Explained Variance	Dominant Correlation Loadings	Physical Interpretation
PC1	4.606/51.18%	$w_{mean}$ 0.977; $w_{SD}$ 0.976; $w_{95}$ 0.970; ${CV}_{w}$ 0.934; ${RMS}_{edge}$ 0.892	Deposition-driven filament expansion and boundary instability
PC2	2.075/23.06%	$\kappa_{mean}$ 0.756; ${RMSE}_{traj}$ 0.739; $\frac{dw}{ds}$ 0.734; ${RMSE}_{curv}$ 0.507	Geometry-dependent tracking and dynamic response
PC3	1.052/11.69%	${RMSE}_{curv}$ 0.776; $\kappa_{mean}$ −0.434; $\frac{dw}{ds}$ −0.275; ${RMSE}_{traj}$ 0.272	Curvature-specific fidelity mode

**Table 3:** Leave-one-out cross-validated model performance; “raw” denotes nozzle diameter, printhead velocity, and categorical trajectory geometry; “+ engineered” additionally includes $A_{dep}$ and $R_{vD}$. Bootstrap uncertainty for the principal raw-input models is reported in the accompanying Section 3.

Model	$R^{2}$	MAE	RMSE	Interpretation
LOOCV training-mean baseline	−0.058	0.457	0.548	Reference predictor; no explanatory structure
Linear Regression (raw)	0.712	0.198	0.286	Parsimonious linear baseline
Ridge Regression (raw)	0.713	0.195	0.285	Regularized primary linear model
Decision Tree (raw)	0.425	0.301	0.404	Shallow nonlinear baseline
Random Forest (raw)	0.541	0.290	0.361	Nonlinear ensemble comparator
Gradient Boosting (raw)	0.709	0.216	0.288	Nonlinear performance comparable to ridge
Ridge Regression (+engineered)	0.730	0.195	0.277	Secondary sensitivity analysis; highest numerical $R^{2}$, but predictors are correlated
Random Forest (+engineered)	0.511	0.308	0.373	Engineered terms did not improve RF performance
Gradient Boosting (+engineered)	0.677	0.234	0.303	Reproduces the original engineered-specification result

**Table 4:** LOOCV permutation importance for the raw controlled predictors. Values are mean increases in out-of-fold RMSE after permutation; larger values indicate greater predictive information. Parentheses indicate descriptive bootstrap 95% intervals.

Raw Predictor	GBR ΔRMSE	Ridge ΔRMSE	Interpretation
Printhead velocity	0.204 (0.128–0.273)	0.221 (0.140–0.293)	Largest or near-largest predictive contribution
Trajectory geometry	0.203 (0.120–0.278)	0.197 (0.120–0.265)	Predictive contribution comparable to printhead velocity
Nozzle diameter	0.141 (0.078–0.200)	0.118 (0.058–0.171)	Smaller but substantive independent contribution

## Data Availability

The construct-level dataset supporting all analyses reported in this study is provided with the submission. Additional raw imaging and ROI-level source data, where available, may be obtained from the corresponding author upon reasonable request.

## Abbreviations

The following abbreviations are used in this manuscript:

CAD	Computer-Aided Design
CV	Coefficient of Variation
EAI	End-Asymmetry Index
EBR	End-Bead Ratio
GBR	Gradient Boosting Regression
LOOCV	Leave-One-Out Cross-Validation
MAE	Mean Absolute Error
ML	Machine Learning
PCA	Principal Component Analysis
PS	Printability Score
RF	Random Forest
RMS	Root Mean Square
RMSE	Root Mean Square Error
ROI	Region of Interest

## Appendix A. Quantitative Variables and Mathematical Definitions

This appendix summarizes the quantitative variables used in the current analysis and provides the mathematical definitions required for reproducibility. Because geometric printability is multidimensional, the workflow intentionally retained complementary descriptors of filament width and width stability, boundary irregularity, trajectory tracking, curvature fidelity, and endpoint behavior rather than relying on a single geometric measure. The composite Printability Score (PS) was therefore treated as a secondary summary index, while interpretation remained anchored to the individual physical descriptors.

All image-derived variables were calculated from standardized top-view microscopy images after segmentation, morphological cleaning, skeletonization, and registration to the corresponding CAD reference trajectory. ROI-level measurements were aggregated to the construct level before PCA and machine-learning analysis.

### Appendix A.1. Summary of Variables

**Table A1:** Summary of Variables.

Variable	Definition and Analytical Role	Unit
$D_{n}$	Nozzle internal diameter; controlled hardware factor	mm
v	Printhead translational velocity; controlled process factor	mm/s
Q	Nominal volumetric delivery rate	mm^3^/s
$A_{dep}$	Nominal lineal deposition load, Q/v	mm^2^
$R_{vD}$	Kinematic speed-nozzle scaling ratio, $v/D_{n}$	s^−1^
G	Reference trajectory geometry; categorical process/geometry factor	categorical
$w_{mean}$	Mean local filament width	µm
$w_{sd}$	Standard deviation of local filament width	µm
$CV_{w}$	Coefficient of variation of local width	dimensionless
$w_{p95}$	95th percentile of local filament width	µm
dw/ds	Longitudinal width gradient along the filament centerline	µm/mm
$RMS_{edge}$	RMS deviation of the measured edge from its smoothed contour	µm
$\bar{\kappa }$	Mean centerline curvature	mm^−1^
$RMSE_{curv}$	RMSE between measured and reference curvature	mm^−1^
$RMSE_{traj}$	Spatial RMSE between measured and reference centerlines	µm
EBR	End-bead ratio; candidate endpoint descriptor defined during workflow development; not available in the final construct-level analysis dataset	dimensionless
EAI	End-asymmetry index; candidate endpoint descriptor defined during workflow development; not available in the final construct-level analysis dataset	dimensionless
PS	Composite study-relative Printability Score	dimensionless

### Appendix A.2. Process-Level Engineering Variables

The nominal lineal deposition load was defined as

$$A_{dep}=\frac{Q}{v}$$

where Q is the nominal volumetric delivery rate (mm^3^/s), and v is the printhead velocity (mm/s). $A_{dep}$ has units of mm^2^ and represents the nominal cross-sectional material load deposited per unit trajectory length.

The kinematic speed-nozzle scaling ratio was defined as

$$R_{vD}=\frac{v}{D_{n}}$$

where $D_{n}$ is the nozzle internal diameter. $R_{vD}$ has units of s^−1^ and is therefore a scaling ratio rather than a dimensionless quantity. Because $A_{dep}$ is a deterministic transformation of speed when Q is held constant and $R_{vD}$ is derived from both speed and nozzle diameter, these variables were interpreted as alternative parameterizations of the controlled process rather than as independent causal factors.

### Appendix A.3. Image-Derived Geometric Descriptors

Local filament widths were obtained from the Euclidean distance transform of the binary filament mask. At skeleton point i, the local width was defined as

$$w_{i}=2d_{i}$$

where $d_{i}$ is the shortest distance from the centerline to the filament boundary. Mean width and its standard deviation were calculated as

$$w_{mean}=\frac{1}{N}\sum_{i=1}^{N}w_{i}$$

$$w_{sd}=\sqrt{\frac{1}{N-1}\sum_{i=1}^{N}{\left(w_{i}-w_{mean}\right)}^{2}}$$

and relative width variability was expressed as

$$CV_{w}=\frac{w_{sd}}{w_{mean}}$$

The 95th-percentile width was defined as

$$w_{p95}=P_{95}\left(w_{i}\right)$$

and was retained as a descriptor sensitive to localized filament widening. Longitudinal width change was estimated by linear regression of width against centerline arc length s,

w(s)=as+b

such that

$$\frac{dw}{ds}=a$$

with units of µm/mm.

Boundary irregularity was quantified as the root-mean-square deviation of the measured edge contour from its smoothed representation:

$$RMS_{edge}=\sqrt{\frac{1}{N}\sum_{i=1}^{N}\delta_{i}^{2}}$$

where $\delta_{i}$ is the local orthogonal deviation between the original and smoothed contours.

For a planar centerline (x(s),y(s)), curvature was defined as

$$\kappa \left(s\right)=\frac{\left|x^{\prime }\left(s\right)y^{\prime \prime }\left(s\right)-y^{\prime }\left(s\right)x^{\prime \prime }\left(s\right)\right|}{{\left(x^{\prime }{\left(s\right)}^{2}+y^{\prime }{\left(s\right)}^{2}\right)}^{3/2}}$$

and mean curvature was defined as

$$\bar{\kappa }=\frac{1}{L}\int_{0}^{L}\kappa \left(s\right)\;ds$$

Curvature fidelity relative to the registered CAD reference trajectory was quantified by

$$RMSE_{curv}=\sqrt{\frac{1}{N}\sum_{i=1}^{N}{\left(\kappa_{i}-\kappa_{i,ref}\right)}^{2}}$$

and positional trajectory fidelity was quantified by

$$RMSE_{traj}=\sqrt{\frac{1}{N}\sum_{i=1}^{N}{∥r_{i}-r_{i,ref}∥}^{2}}$$

Endpoint metrics were defined during workflow development as the end-bead ratio (EBR) and end-asymmetry index (EAI), describing terminal widening and start–end asymmetry. However, their construct-level values were not available in the locked analytical dataset, and they were therefore excluded from the reported PS, correlation analysis, PCA, and predictive modeling. They are retained in the Appendix A only to document the broader candidate workflow.

### Appendix A.4. Composite Printability Score and Modeling Target

Four complementary instability metrics were used to construct the composite Printability Score: $CV_{w}$, $RMS_{edge}$, $RMSE_{traj}$, and $RMSE_{curv}$. Each variable was min–max-normalized within the study dataset:

$$x_{i}^{*}=\frac{x_{i}-x_{min}}{x_{max}-x_{min}}$$

and the normalized components were combined with equal weighting:

$$PS=CV_{w}^{*}+RMS_{edge}^{*}+RMSE_{traj}^{*}+RMSE_{curv}^{*}$$

Higher PS values indicate poorer geometric printability. Because normalization is relative to the present dataset, PS is a study-specific comparative index rather than a universal printability scale.

For supervised prediction, the primary input vector comprised the raw controlled process variables and categorical trajectory geometry:

$$x_{raw}\;=\;[D_{n},\;v,\;G]$$

where G denotes trajectory geometry represented by one-hot encoding. A secondary engineering specification added the deterministic reparameterizations $A_{dep}$ and $R_{vD}$:

$$x_{eng}\;=\;[D_{n},\;v,\;A_{dep},\;R_{vD},\;G]$$

Image-derived variables used to construct PS were not included as direct predictors. The raw-input specification was used for primary model interpretation, whereas the engineered specification was treated as a secondary sensitivity analysis because $A_{dep}$ and $R_{vD}$ are deterministic transformations of the controlled settings. Linear Regression, Ridge Regression, and a shallow Decision Tree were included as simple baselines/comparators alongside RF and GBR under the same LOOCV framework.
